# Integrated Metabolomic–Transcriptomic Analyses of Flavonoid Accumulation in Citrus Fruit under Exogenous Melatonin Treatment

**DOI:** 10.3390/ijms25126632

**Published:** 2024-06-16

**Authors:** Chenning Zhao, Zhendong Wang, Zhenkun Liao, Xiaojuan Liu, Yujia Li, Chenwen Zhou, Cui Sun, Yue Wang, Jinping Cao, Chongde Sun

**Affiliations:** 1 Laboratory of Fruit Quality Biology, Zhejiang Provincial Key Laboratory of Horticultural Plant Integrative Biology, The State Agriculture Ministry Laboratory of Horticultural Plant Growth, Development and Quality Improvement, Zhejiang University, Hangzhou 310058, China; chenningzhao@zju.edu.cn (C.Z.); wfkwwzd@163.com (Z.W.); liaozhenkun@zju.edu.cn (Z.L.); yujiali0201@163.com (Y.L.); 12116046@zju.edu.cn (C.Z.); fruit@zju.edu.cn (Y.W.); caojinpingabc@126.com (J.C.); 2 State Key Laboratory of Subtropical Silviculture, College of Forestry and Biotechnology, Zhejiang A&F University, Hangzhou 311300, China; xiaojuanliu2020@163.com; 3 Hainan Institute, Zhejiang University, Sanya 572000, China; suncui7@126.com

**Keywords:** citrus, flavonoids, melatonin, regulation, metabolome, transcriptome

## Abstract

The flavonoids in citrus fruits are crucial physiological regulators and natural bioactive products of high pharmaceutical value. Melatonin is a pleiotropic hormone that can regulate plant morphogenesis and stress resistance and alter the accumulation of flavonoids in these processes. However, the direct effect of melatonin on citrus flavonoids remains unclear. In this study, nontargeted metabolomics and transcriptomics were utilized to reveal how exogenous melatonin affects flavonoid biosynthesis in “Bingtangcheng” citrus fruits. The melatonin treatment at 0.1 mmol L^−1^ significantly increased the contents of seven polymethoxylated flavones (PMFs) and up-regulated a series of flavonoid pathway genes, including *4CL* (4-coumaroyl CoA ligase), *FNS* (flavone synthase), and *FHs* (flavonoid hydroxylases). Meanwhile, *CHS* (chalcone synthase) was down-regulated, causing a decrease in the content of most flavonoid glycosides. Pearson correlation analysis obtained 21 transcription factors co-expressed with differentially accumulated flavonoids, among which the AP2/EREBP members were the most numerous. Additionally, circadian rhythm and photosynthesis pathways were enriched in the DEG (differentially expressed gene) analysis, suggesting that melatonin might also mediate changes in the flavonoid biosynthesis pathway by affecting the fruit’s circadian rhythm. These results provide valuable information for further exploration of the molecular mechanisms through which melatonin regulates citrus fruit metabolism.

## 1. Introduction

Citrus, a globally cultivated fruit crop, is highly favored by consumers for its sour-sweet taste, intense aroma, refreshing juice, and dense nutrition. In traditional Chinese medicine, many tissues of citrus fruits serve as essential sources for traditional herbal medicine, such as “Chenpi”, the dried peel of citrus fruits [1]. Modern medical research has proved that citrus fruit extracts possess diverse therapeutic properties, such as anticancer, antitumor, and anti-inflammation activities and cardiovascular disease prevention [2]. These health-beneficial properties are believed to be attributed to the rich content of natural products in fruits, including vitamins, carotenoids, and especially flavonoids [3]. 

Flavonoids are among the most predominant bioactive substances in citrus and accumulate in large quantities in the peel and pulp. However, the types and abundance of flavonoids vary across different citrus species and parts [4,5]. Flavanone glycosides and polymethoxylated flavones (PMFs) are the two main classes of flavonoids in citrus. The former is distributed in the pulp and peel of almost all citrus species, whereas the latter is exclusively present in the peel of fruits from *Citrus sinensis*, *Citrus reticulata*, and related hybrids [6]. PMFs are flavones bearing multiple methoxy groups, which endows them with low polarity, poor water solubility, and strong cell membrane permeability [7]. PMFs exhibit more robust bioactivities [8] and higher potential for medicinal development, gradually becoming star molecules in plant natural products. Nevertheless, studies on the biological regulation of characteristic citrus flavonoids remain limited so far. Exploring effective treatments to enhance the accumulation of citrus flavonoids, particularly PMFs, will be of great significance to their application.

Flavonoids also serve as important regulators involved in various physiological processes such as growth and development, morphogenesis, light response, and stress resistance [9,10]. Correspondingly, changes in environmental factors or hormones can affect the accumulation of plant flavonoids [11]. Melatonin (N-acetyl-5-methoxytryptamine), an indole compound, is a highly evolutionarily conserved endogenous molecule in eukaryotes. Since its discovery in higher plants in 1995, its diverse function in plants has been extensively studied [12]. Wei et al. [13] identified the plant melatonin receptor protein CAND2/PMTR1 in *Arabidopsis thaliana*, which further confirmed melatonin as an indole phytohormone. Numerous investigations have shown that melatonin possesses superior antioxidant capacity, including scavenging ROS and RNS (reactive oxygen or nitrogen species) generated by plants under adversity and regulating the expression of redox enzymes, thus helping plants to resist various biotic or abiotic stresses [14]. Melatonin can also participate in the regulation of the metabolism of other plant hormones through crosstalk [15,16] and act as a regulator of plant circadian rhythms and photosynthesis [17], thereby influencing plant growth and morphogenesis [18].

Many studies have reported that melatonin regulation of plant stress tolerance or metabolic processes is also accompanied by changes in flavonoids [19], thus altering the physiological qualities of fruits or vegetables. For example, exogenous melatonin can enhance the biosynthesis of flavonols and flavanols in grape (*Vitis vinifera*) fruits [20], promote the accumulation of flavonoids by regulating transcription factors, and affect DNA methylation in grapes [21]. The content of flavonoids in kiwifruit (*Actinidia Chinensis*) leaves increased after melatonin treatment, and a series of flavonoid pathway genes, including *CHS* (chalcone synthase), *F3H* (flavonoid 3-hydroxylase), and *FNS* (flavone synthase), were also up-regulated [22]. Preharvest melatonin treatment of pear (*Pyrus bretschneideri*) fruits can increase the content of anthocyanins in fruit peel during ripening [23]. Moreover, in fresh jujube (*Zizyphus jujuba*), exogenous melatonin can increase the level of phenolic compounds and up-regulate *PAL* (phenylalanine ammonia-lyase), *CHS*, *F3H*, etc. [24]. However, there are also some contradictory results. A study on fresh-cut lotus root (*Nelumbo nucifera* Gaertn.) found that melatonin reduced the flavonoid content and decreased enzyme activities of the flavonoid pathway, thereby slowing down the browning [25]. Cold stress induces anthocyanin synthesis in plum (*Prunus salicina* Lindl.) fruits, but melatonin treatment can effectively inhibit the increase in anthocyanin caused by cold stress and down-regulate several related transcription factors [26]. These diverse findings suggest that the regulatory effects of melatonin on flavonoids might be closely related to factors such as plant species, type of flavonoids, treatment methods, and environmental conditions. However, it is still unclear whether melatonin directly influences the biosynthesis of citrus flavonoids and which kinds of flavonoids are mainly regulated.

The biosynthesis of plant flavonoids originates from the phenylpropanoid metabolic pathway. Currently, the biosynthesis pathway of citrus flavonoids has been deciphered, and our previous studies have identified and functionally characterized a range of critical genes, such as *CHS*, *CHI* (chalcone isomerase), *FNS*, *F3’H* (flavonoid 3’-hydroxylase), and *FOMTs* (flavonoid *O*-methyltransferases) [27,28,29,30,31,32]. However, the regulatory mechanisms of citrus flavonoids in different physiological processes still lack systematic and in-depth studies. This study focused on the impact and regulation of melatonin on citrus fruit flavonoids. Metabolomic and transcriptomic approaches were utilized to explore and decipher the possible molecular mechanisms and metabolic basis of melatonin’s influence on the biosynthesis of citrus flavonoids. The results show that melatonin affected flavonoid content at a specific concentration (0.1 mmol L^−1^), with PMFs and other flavonoids exhibiting distinct trends after treatment. Many genes in the phenylpropanoid metabolism pathway were differentially expressed, including *CHS* and *FNS*. Additionally, multiple *AP2-EREBP* transcription factors (TFs) exhibited a melatonin response and co-expression with flavonoids, while circadian rhythm-related pathways also underwent significant changes. The integration of multi-omics data provided valuable insights into the global changes induces by melatonin in citrus fruit peel, thereby contributing to a better understanding of the regulatory mechanisms of citrus flavonoids.

## 2. Results

### 2.1. Effect of Exogenous Melatonin Treatments on Citrus Flavonoid Content

**Figure 1:**
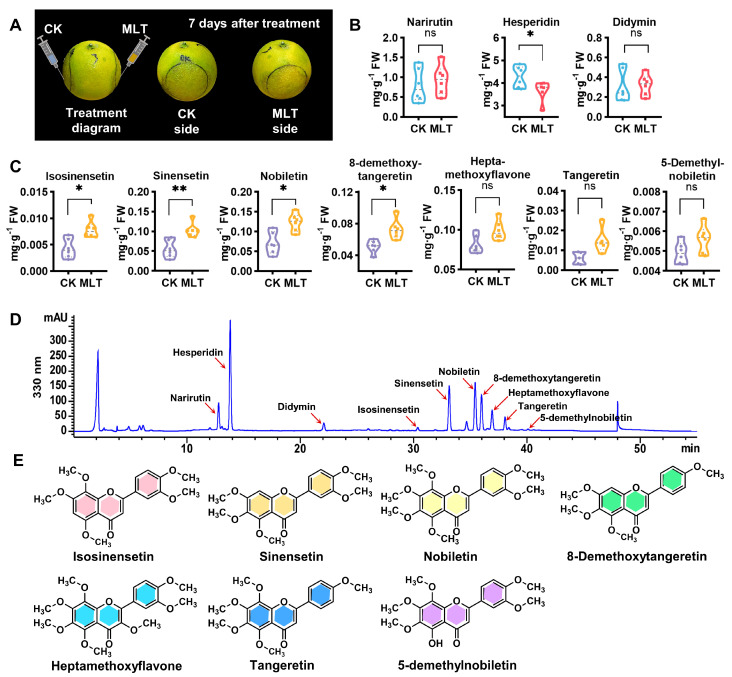
Illustrations of the melatonin treatment and effects of 0.1 mmol L^−1^ melatonin on citrus fruit flavonoid contents. (**A**) The experiment design and sample chart of melatonin treatment on citrus fruit. (**B**,**C**) Changes in individual FG (flavanone glycoside) contents (**B**) and individual PMF (polymethoxylated flavone) contents (**C**) after 0.1 mmol L^−1^ melatonin treatment. CK, control group; MLT, melatonin treatment group; FW, fresh weight. Error bars represent SE (*n* = 6). Statistical analysis: paired Student’s *t*-test (* *p* < 0.05; ** *p* < 0.01). (**D**) The representative HPLC profile of flavonoids detected in “Bingtangcheng” fruit peel at 330 nm. (**E**) Structures of 7 representative PMFs.

Citrus fruits have thick wax and cuticle, which may affect the absorption efficiency of sprayed or soaked hormones on fruit. In order to observe the effects of exogenous melatonin on flavonoid biosynthesis more directly, the injection method was used for treatment, as shown in Figure 1A. Ten flavonoids, including three flavanone glycosides and seven PMFs, were identified from “Bingtangcheng” fruit peel by using the high-performance liquid chromatography (HPLC) method (Figure 1B–D). Four gradient concentrations were selected for treatment to determine the optimal melatonin dose affecting flavonoid metabolism. The results demonstrate that melatonin at a concentration of 0.1 mmol L^−1^ had a significant effect on citrus flavonoids, especially polymethoxylated flavones (PMFs), while the impact was not pronounced at other concentrations (Figure S1). Further analysis of the changes in individual flavonoids after 0.1 mmol L^−1^ melatonin treatment revealed that hesperidin, which accounted for the highest proportion of total flavonoids, decreased sharply by 14.34%, yet the other flavanone glycosides did not change a lot (Figure 1B). Meanwhile, the contents of all PMFs increased, among which four PMFs (isosinensetin, sinensetin, nobiletin, and 8-demethoxytangeretin) increased significantly (Figure 1C,E). Sinensetin and nobiletin, the PMFs with higher contents, increased by 85.46% and 74.56%, respectively. This indicates that the effects of melatonin on citrus flavonoid biosynthesis require specific concentrations and that PMF accumulation was evidently induced by exogenous melatonin.

### 2.2. Global Changes in Metabolome of Citrus Peel after 0.1 mmol L^−1^ Melatonin Treatment

**Figure 2:**
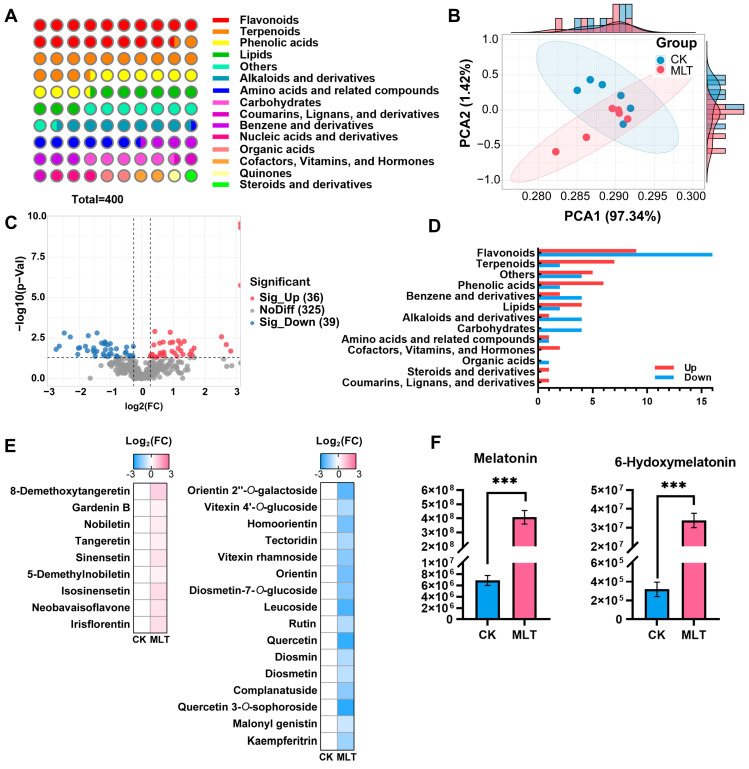
Metabolomic analyses of citrus fruit under 0.1 mmol L^−1^ melatonin treatment. (**A**) Classification of identified metabolites in citrus samples. (**B**) Principal component analysis (PCA) of all metabolites in control (CK) and 0.1 mmol L^−1^ melatonin treatment (MLT) groups. (**C**) Volcano plot of differentially accumulated metabolites (DAMs) in comparison group “CK vs. MLT”. (**D**) Classification and statistics of up- or down-regulated DAMs. (**E**) Specific substances of flavonoid DAMs and their fold changes in comparison group “CK vs. MLT”. (**F**) Abundance changes in melatonin and 6-hydroxymelatonin detected in metabolome between CK and MLT groups. Error bars represent SE (*n* = 6). Statistical analysis: paired Student’s *t*-test (*** *p* < 0.001).

Metabolomic and transcriptomic analyses were conducted on samples treated with 0.01 mmol L^−1^ melatonin to explore the specific molecular mechanisms underlying the flavonoid content changes caused by exogenous melatonin. Over 5000 substances were detected in the nontargeted metabolomics by using ultra-performance liquid chromatography (UPLC)-MS/MS, among which 400 substances were identified. They could be classified into 15 categories, with flavonoids and terpenoids being the most abundant, followed by phenylpropanoids (Figure 2A and Table S1). Principal component analysis (PCA) based on 400 metabolites reflected the distribution of 12 samples and the separation trend among the samples. PC1 and PC2 explained 97.34% and 1.42% of metabolite variances among different samples, respectively (Figure 2B). The samples in the CK (control) group were relatively aggregated, while those in the MLT (melatonin treatment) group were more discrete. Despite the overlap of the confidence ellipses between the two groups of samples, they could still be separated by PC2. 

The volcano plot displays the distribution of differentially accumulated metabolites (DAMs) between the CK and MLT groups. After melatonin treatment, 36 metabolites were significantly up-regulated, and 39 were significantly down-regulated (Figure 2C). The classification and the number statistics of DAMs are shown in Figure 2D, with flavonoids and terpenoids having the highest number of metabolites. The DAMs of flavonoids were predominantly down-regulated, and those of terpenoids were mainly up-regulated. A detailed analysis of the changes in 25 differentially accumulated flavonoids (DAFs) revealed that 7 of the 9 up-regulated flavonoids were PMFs. In contrast, the 16 down-regulated flavonoids were mainly flavonol or flavone glycosides without PMFs (Figure 2E). This result was consistent with the quantitative analysis by HPLC in Figure 1, which further confirmed that the PMFs had different trends from other flavonoids after melatonin treatment. Melatonin and its metabolic product 6-hydroxymelatonin were detected in the metabolome and were significantly elevated, demonstrating the successful modeling of melatonin treatment (Figure 2F). 

### 2.3. Global Changes in Transcriptome of Citrus Fruit Peel after 0.1 mmol L^−1^ Melatonin Treatment

**Figure 3:**
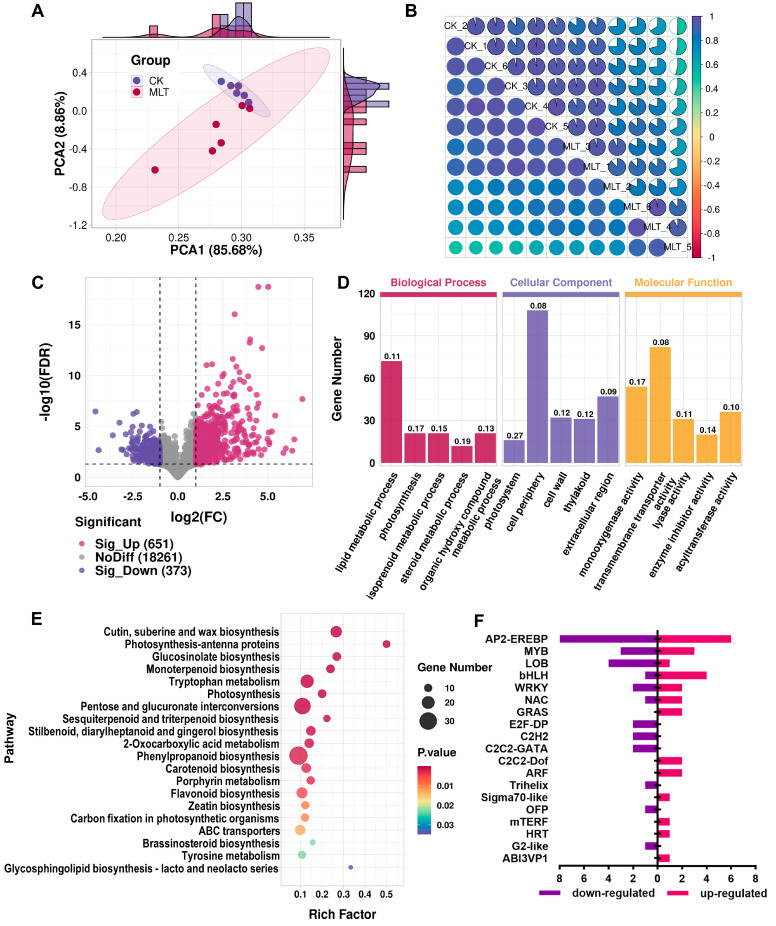
Transcriptomic analyses of citrus fruit under 0.1 mmol L^−1^ melatonin treatment. (**A**) PCA score plot of transcriptomic data from the control (CK) and 0.1 mmol L^−1^ melatonin treatment (MLT) groups. (**B**) Pearson correlation analysis of the transcriptomic data among samples. The color gradient indicates the correlation coefficients from positive (dark blue) to negative (crimson). (**C**) Volcano plot of the differentially expressed genes (DEGs) in comparison group “CK vs. MLT”. (**D**) GO annotation enrichment of DEGs. The top 5 terms (sorted by *p*-value) in each category are displayed. The numerals above the columns represent the rich factors. (**E**) KEGG pathway enrichment of DEGs. The top 20 pathways (sorted by *p*-value) are displayed. (**F**) Statistics of differentially expressed transcription factors (TFs).

The two groups of samples were further analyzed for changes at the transcriptional level by RNA sequencing. A total of 22,482 genes were detected in the 12 samples, including 1105 new genes. PCA analysis revealed that PC1 and PC2 could explain 85.68% and 8.86% of the transcript variances among samples, respectively (Figure 3A). Consistent with the PCA analysis of the metabolomic data, the transcriptomic data of the CK samples were significantly clustered. In contrast, the MLT samples were more dispersed, suggesting considerable individual variations in the effects of the melatonin treatment on different fruits. The confidence ellipses of the two groups of samples overlapped, but they could be separated by the PC2 dimension. The correlation analysis also showed that the samples from the CK group highly correlated with each other, but the sample correlations in the MLT group were uneven (Figure 3B). 

Differentially expressed genes (DEGs) between CK and MLT groups were obtained with the cutoff threshold of |log_2_(fold change)| ≥ 1 and false discovery rate (FDR) < 0.05 and visualized by a volcano plot (Figure 3C). After melatonin treatment, 651 genes were significantly up-regulated, and 373 were significantly down-regulated. Most DEGs’ log_2_(FC) values ranged from −2.5 to 2.5. Enrichment of DEGs based on function and annotation is an effective way to understand changes in molecular pathways. The results of GO term enrichment showed that in the “Biological Process” category, the DEGs of the term “lipid metabolic process” were the most numerous, and the term “photosynthesis” and “isoprenoid metabolic process” were also significantly enriched. In the “Cellular Component” category, the term “photosystem” had the highest enrichment significance and rich factor. In the “Molecular Function” category, the terms “transmembrane transporter activity”, “monooxygenase activity”, and “acyltransferase activity” included the most DEGs (Figure 3D). The top 20 KEGG pathways (all with *p*-value < 0.5) with the highest enrichment significance are displayed in the bubble plot. As shown in Figure 3E, the phenylpropanoid biosynthesis pathway obtained the most DEGs, and the flavonoid biosynthesis pathway was also significantly enriched. In addition, three pathways related to photosynthesis were significantly enriched: “photosynthesis-antenna proteins”, “photosynthesis”, and “carbon fixation in photosynthetic organisms”. Four relevant pathways in the terpenoid metabolism were also enriched: “monoterpenoid biosynthesis”, “sesquiterpenoid and triterpenoid biosynthesis”, “carotenoid biosynthesis”, and “zeatin biosynthesis”. Except for the pathway genes, 56 differentially expressed TFs were categorized and counted (Figure 3F and Table S2). It was found that the AP2-EREBP family had the highest number of DEGs, followed by the MYB, LOB, and bHLH families. The numbers of TFs down-regulated and up-regulated were roughly the same.

### 2.4. Combined Analysis of DAMs and DEGs Involved in Phenylpropanoid and Flavonoid Biosynthesis Pathway

**Figure 4:**
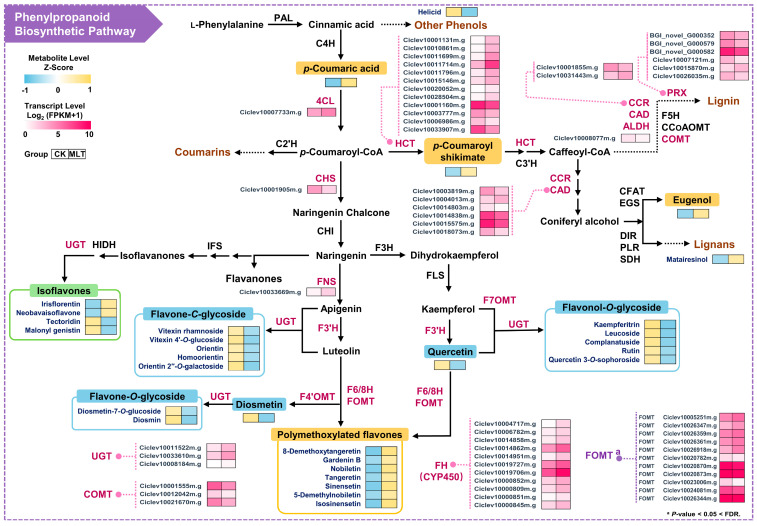
Combined analysis of DEGs and DAMs in phenylpropanoid and flavonoid metabolism pathways. The color gradient from blue to yellow indicates the metabolite levels from low to high. The color gradient from white to pink indicates the relative transcript expression levels from low to high. Data are means of six biological replicates in each group. The enzyme names in pink indicate the presence of DEGs in the coding genes. The metabolite names with yellow/blue/green background indicate that the substances are up-regulated/down-regulated/both, respectively. Dash lines and metabolites in brown color indicate access to another metabolism pathway.

Combining the phenylpropanoid biosynthetic pathway and related branches, a conjoint analysis of the metabolic flow of phenylpropanoids and the distribution of DEGs in the pathway was conducted (Figure 4 and Table S3). *p*-Coumaroyl-CoA is an essential precursor of multiple branches, and its precursor, *p*-coumaric acid, was significantly up-regulated after melatonin treatment. The corresponding synthase 4CL (4-coumaroyl-CoA ligase) also showed a significant increase in the expression level of one encoding gene. HCT (shikimate *O*-hydroxycinnamoyl transferase), a key enzyme catalyzing the entry of *p*-coumaroyl-CoA into the lignin biosynthetic pathway, had 12 encoding genes differentially expressed, of which 8 were up-regulated and 4 down-regulated. This agreed with the significant increase in the abundance of the counterpart product, *p*-coumaroyl shikimate. Several genes in the lignin pathway, such as *CAD* (cinnamyl alcohol dehydrogenase), *CCR* (cinnamoyl-CoA reductase), *ALDH* (coniferyl-aldehyde dehydrogenase), *COMT* (caffeic acid *O*-methyltransferase), and *PRX* (peroxidase), were also differentially expressed, but with varying trends. The abundance of eugenol and matairesinol, which are synthesized in the lignin-related pathway, also increased. These results suggest that more metabolic flux from the phenylpropanoid pathway enters the lignin biosynthesis branch. 

As a competitive pathway of lignin biosynthesis, flavonoids were synthesized from *p*-coumaroyl-CoA through CHS (chalcone synthase) catalysis. After melatonin treatment, the *CHS* gene was significantly down-regulated (Figure 4), consistent with decreased flavanone glycoside and flavonol glycoside contents. The *FNS* (flavone synthase) gene was significantly up-regulated, causing the increase in all PMFs’ abundance. But the flavone glycoside content decreased, which might be closely related to changes in the expression of other modifying enzymes. The biosynthesis pathway of PMFs involves multiple steps of hydroxylation and methylation modification, but many *FHs* (flavonoid hydroxylases) and *FOMTs* (flavonoid *O*-methyltransferases) have yet to be identified. Based on the gene family analysis and phylogenetic clustering in our previous studies [28,31], the potential differentially expressed *FH* and *FOMT* genes (listed in Table S3) from the *CYP450* and *OMT* families were selected and analyzed. Interestingly, 11 differentially expressed *FHs* were significantly up-regulated, but no other *OMT* genes met the threshold of DEGs except for the 3 *COMTs* in the lignin pathway (Figure 4). The screening thresholds of *FOMTs* were further expanded to a fold change > 2 or < 0.5 and *p* < 0.05 < FDR, and the results show that 11 potential *FOMTs* were differentially expressed, of which 5 were up-regulated and 6 were down-regulated. Taken together, it was speculated that the increment in PMF abundances might be associated with the collective up-regulation of *FNS*, *FHs*, and some *FOMTs*.

### 2.5. Screening of Candidate TFs Related to Flavonoid Changes

**Figure 5:**
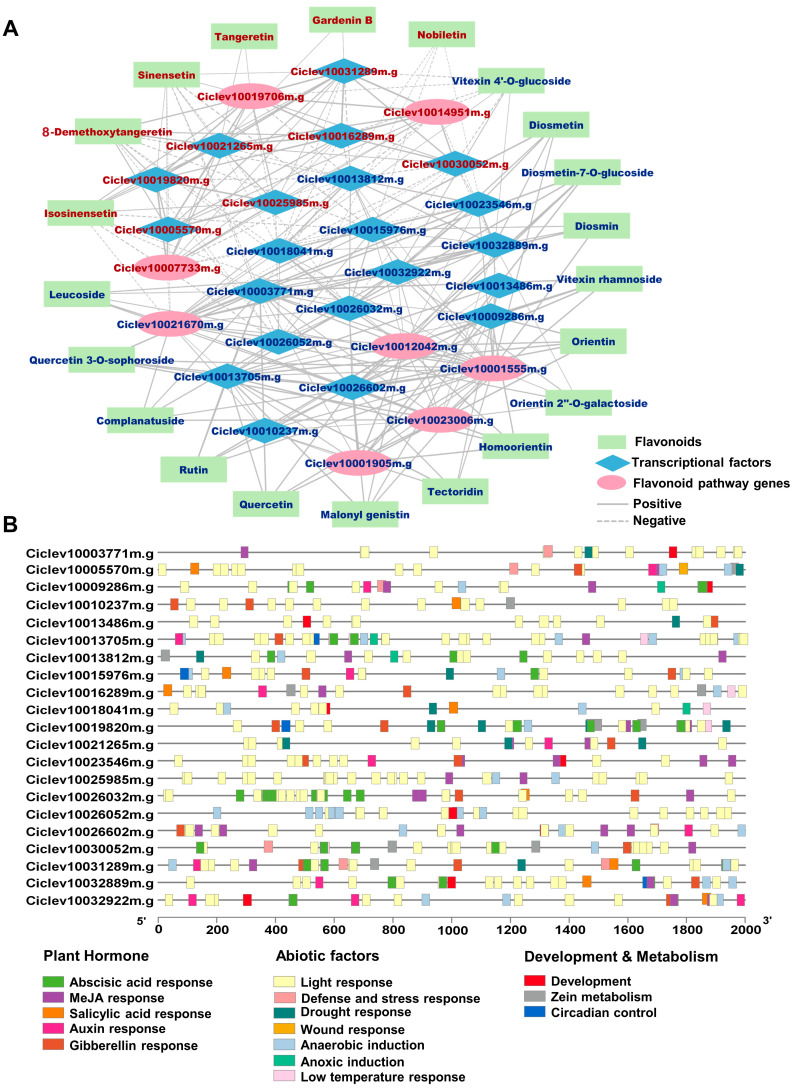
Transcription factors co-expressed with differentially accumulated flavonoids. (**A**) Co-expression network of DAFs, DEGs in flavonoid pathway, and differentially expressed TFs. Up-regulated genes or metabolites are shown in crimson fonts, while down-regulated ones are in dark blue. The higher the correlation coefficient, the thicker the connection line. (**B**) Promoter cis-elements analysis of TFs co-expressing with DAFs.

A co-expression network was established to explore TFs correlated with DAMs and DEGs in the flavonoid pathway, thereby identifying potential TFs that might respond to the melatonin signal and participate in flavonoid biosynthesis. As shown in Figure 5A, the abundance of DAFs was highly correlated with the expression patterns of 21 flavonoid pathway DEGs and TFs, among which 7 were up-regulated and 14 were down-regulated. The specific information of the 21 TFs is listed in Table 1. A total of 8 AP2/EREBP members were found in the highest proportion of the 21 TFs. The transcript expression level of *Ciclev10021265m.g* increased the most after melatonin treatment, while *Ciclev10023546m.g* showed the highest fold decrease in expression. Research suggests that melatonin could regulate gene expression via plant hormone signaling or transduction pathways [15], so the promoter cis-elements of the 21 TFs were further analyzed (Figure 5B). It was found that all candidate TFs contained hormone response elements in their promoters (Table 1), and the number of hormone response elements in the promoter of *Ciclev10026032m.g* and *Ciclev10031289m.g* was greater than or equal to 20. In addition, three TFs, *Ciclev10015976m.g*, *Ciclev10019820m.g*, and *Ciclev10032889m.g*, included circadian control elements in their promoters, suggesting that their expression might also be under regulation by the circadian rhythm.

### 2.6. Response of Citrus Circadian Oscillator and Photosynthesis System to Exogenous Melatonin Treatment

**Figure 6:**
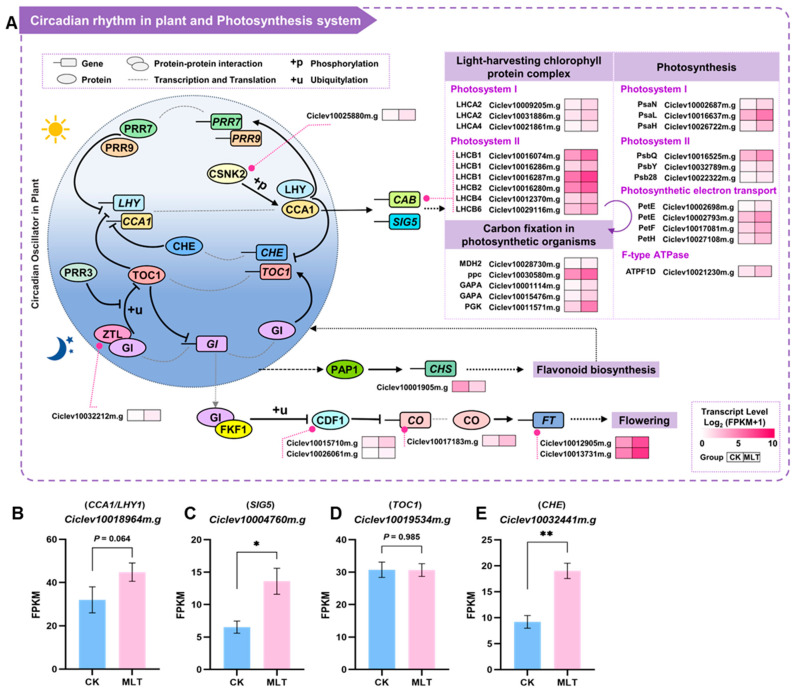
Analysis of DEGs in circadian oscillator and photosynthesis systems in citrus fruit after 0.1 mmol L^−1^ melatonin treatment. (**A**) Heatmap of DEGs in circadian oscillator and photosynthesis system. The color gradient from white to pink indicates the relative transcript expression levels from low to high. Data are means of six biological replicates in each group. (**B**–**E**) The transcript expression levels of the predicted circadian-related genes in citrus. Error bars represent SE (*n* = 6). Statistical analysis: paired Student’s *t*-test (* *p* < 0.05; ** *p* < 0.01).

Notably, both GO and KEGG enrichment analyses showed significantly enriched genes in photosynthesis (Figure 3D,E). It is well known that the flavonoid biosynthesis pathway and the photosynthesis system are both regulated by the plant’s circadian rhythm [33,34]. Given the association between melatonin and plant circadian rhythm, the DEGs in the circadian rhythm and photosynthesis system were comprehensively analyzed (Figure 6, Table S4). The circadian oscillator is a feedback loop composed of several core proteins that enable rhythmic gene expression through transcriptional and post-translational modification processes such as activation, repression, ubiquitination, and phosphorylation [35]. As shown in Figure 6A, *CSNK2* (casein kinase II subunit alpha) and *ZTL* (ZEITLUPE) in the core oscillator were up-regulated after melatonin treatment. CSNK2 can activate CCA1 (CIRCADIAN CLOCK ASSOCIATED 1) through phosphorylation and promote the binding of CCA1 to DNA, thus altering the rhythmic cycle of plants. And ZTL can degrade TOC1 (TIMING OF CAB EXPRESSION1) through ubiquitination, while TOC1 and CCA1 are mutual expression deterrents. Therefore, the up-regulation of *CSNK2* and *ZTL* led to an increase in the protein activity of CCA1, which can activate *CABs* (chlorophyll a/b-binding proteins) or *SIG5* (SIGMA FACTOR 5), thereby up-regulating the expression of the light-harvesting chlorophyll protein complex genes. The homologs of the Arabidopsis *CCA1*, *LHY1* (LATE ELONGATED HYPOCOTYL), *CHE* (CCA1 HIKING EXPEDITION), *TOC1*, and *SIG5* genes in the citrus genome were searched via protein BLAST. Four genes with the highest identity were selected, of which Ciclev10018964m.g was highly homologous to both CCA1 and LHY1, with 40.33% and 46.08% identity, respectively. The transcription levels of these four genes are shown in Figure 6B–E. Except for *Ciclev10019536m.g* (homolog of *TOC1*), the others were all up-regulated, and the expression levels of the *SIG5* and *CHE* homologs were significantly increased at a statistical level. These results confirm the melatonin regulation of the citrus circadian rhythm.

There was a total of 20 DEGs in the photosynthesis system involving six protein complexes, and all of them were significantly up-regulated (Figure 6A). Changes in photosynthesis system genes also affects subsequent carbon fixation. It was found that the expression level of five genes in the carbon fixation system was significantly elevated in citrus after melatonin treatment, suggesting that exogenous melatonin might affect photosynthesis in citrus through the circadian rhythm. Except for the core oscillator, the circadian clock also has some output signaling pathway, such as flowering time and flavonoid biosynthesis, that was regulated by the oscillator. The expression levels of the *CDF1* (*CYCLING DOF FACTOR 1*), *CO* (*CONSTANS*), and *FT* (*FLOWERING LOCUS T*) genes in the flowering signaling pathway increased (Figure 6A, Table S4), which might be related to circadian rhythm disorder. Meanwhile, this disorder led to the down-regulation of the *CHS* expression level and even the decrease in total flavonoid contents.

### 2.7. RT-qPCR Validation

**Figure 7:**
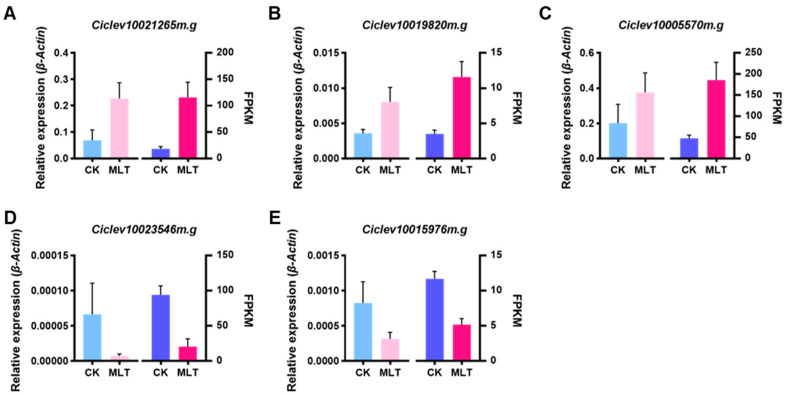
Expression profiles of the candidate transcription factors verified by RT-qPCR. (**A**) Ciclev10021265m.g, (**C**) Ciclev10005570m.g, and (**D**) Ciclev10023546m.g are AP2-EREBP family members. (**B**) Ciclev10019820m.g and (**E**) Ciclev 10015976m.g belong to WRKY and NAC, respectively. The information on the five TFs is listed in Table 1. In each sub-figure, the left panel showed the gene relative expression levels detected by RT-qPCR, and the right panel showed the gene FPKM values obtained by RNA-seq. Error bars represent SE (*n* = 6).

To confirm the reliability of the RNA-seq data, five members with the highest expression levels from the candidate TFs listed in Table 1 were selected for RT-qPCR analysis. The results show that the relative expression patterns of these TFs were consistent with the trends demonstrated by the RNA-seq data (Figure 7), indicating that the RNA-seq data are accurate and credible.

## 3. Discussion

Both melatonin and flavonoids are superior antioxidants. Some adverse environmental factors can induce the synthesis of flavonoids [36], while melatonin can mitigate the impacts of adversity on plants [14]. Numerous studies have shown that melatonin treatment might change the accumulation of flavonoids or other phenolic compounds. However, for different plants and flavonoid classes, melatonin seems to show differential regulatory roles [22,26], and there is a specific dose effect [37]. By using different treatment methods and concentrations of melatonin in sweet cherries (*Prunus avium*), the changes in anthocyanin content varied drastically [38,39]. In this study, citrus fruits were treated with four different concentrations of melatonin, and only the 0.1 mmol L^−1^ concentration had a significant effect on flavonoids (Figure S1 and Figure 1B,C). This is a typical concentration used when treating fruit with melatonin [37], and the use of this concentration has been shown to increase flavonoids in postharvest treatments of grapes [40] and strawberries [41]. However, the present study found that the total flavonoid content decreased after 0.1 mmol L^−1^ melatonin treatment, and the abundance of flavanone glycosides, flavone glycosides, and flavonol glycosides decreased significantly. Conversely, the content of PMFs increased (Figure 1C and Figure 2E). A series of genes in the flavonoid pathway showed differential expression in different patterns. Combined analysis suggested that the decrease in total flavonoids here was closely related to the down-regulation of *CHS*, the rate-limiting enzyme in the flavonoid pathway. The up-regulation of multiple *HCTs* in the lignin pathway, which competed for the common substrate *p*-coumaroyl CoA, might also reduce total flavonoid content (Figure 4; Table S1). The differential changes in flavonoid glycosides and PMFs may be more related to their different synthesis branches and chemical properties. For example, in sweet cherries, treatment with melatonin resulted in an increase in total phenol content but a decrease in total flavonoid and anthocyanin contents [39]. As a major flavonoid component in citrus peel, the increase in PMF content was consistent with the up-regulation of the *FNS* gene. Moreover, the synthesis of PMFs involved multi-step hydroxylation and *O*-methylation, and transcriptome analysis revealed the up-regulation of most potential *FH*s, which may be associated with the increase in PMFs (Figure 4; Table S3). This was also consistent with the results of Song et al. [19]. They found that melatonin could significantly up-regulate *F3’H* to promote the synthesis of luteolin, thereby enhancing salt stress resistance in pigeon peas (*Cajanus cajan*).

COMT can both participate in flavonoid *O*-methylation and catalyze the methylation of *N*-acetylserotonin in the melatonin pathway [42], which links melatonin synthesis to the flavonoid pathway. Many investigations have shown that endogenous melatonin and flavonoids have a reciprocal inhibitory effect. Overexpressing *FLS* (flavonol synthase) in rice also led to an increase in flavonol content and a decrease in melatonin content. Both caffeic acid and quercetin could significantly inhibit the catalyzation of *N*-acetylserotonin by OsCOMT protein in vitro [43]. Overexpressing *MsASMT* (acetylserotonin-*O*-methyltransferase) in alfalfa (*Medicago sativa* L.) also led to an increase in endogenous melatonin and a decrease in flavonoids such as quercetin and rutin [44]. In the present study, exogenous melatonin was found to inhibit the expression of *TDC* (l-tryptophan decarboxylase) and three *COMTs* but to up-regulate the expression of *ASMTs* (Table S5), implying that the flavonoid and melatonin pathways were synergistically regulated in response to exogenous melatonin signaling, causing differential changes in PMFs and other flavonoids. The increase in PMF content might result from the up-regulation of *FNS* and *FHs*, while the decrease in other flavonoid glycosides might be directly related to the inhibition of the melatonin and the down-regulation of *CHS*. Moreover, several critical genes of the flavonoid biosynthesis pathway have been identified in previous studies. However, the expression changes in these genes did not meet the DEG screening threshold in this study. RT-qPCR analysis accompanied by transcriptomic data revealed that the expression levels of nine previously reported genes showed similar change trends to that of DEGs from the same gene family (Figure S2). This result suggested that the expression alteration in flavonoid pathway genes induced by melatonin was universal.

According to co-expression network analysis, several TFs were identified highly associated with DAFs, among which the AP2-EREBP family members were predominant, of which three were significantly up-regulated and five down-regulated (Table 1). The two TFs with the highest up- and down-regulation fold changes belonged to this family. AP2/EREBP TFs can respond to hormonal signals such as ethylene and thus regulate various physiological processes in plants. Previous research discovered that three AP2/EREBP factors could activate flavonoid biosynthesis during citrus development [29]. However, whether AP2/EREBP in citrus responds to melatonin signals remains unclear. Yue et al. [45] found that melatonin could mitigate the inhibitory effect of BrERF2/BrERF09 on the *FLS* promoter, thereby promoting flavonoid synthesis in flowering Chinese cabbage (*Brassica rapa* var. parachinensis). Melatonin treatment can alleviate kiwifruit softening by slowing ethylene release and suppressing the production of acetaldehyde and ethanol to prolong the shelf life. AdERF5, AdERF6, and AdERF75 might be involved in this process [46]. Our results revealed that besides inducing the differential expression of numerous AP2/EREBP TFs, melatonin treatment also caused an increase in jasmonic acid and abscisic acid (ABA) contents, as well as the up-regulation of the key ABA synthesis gene, *NCED*s (9-*cis*-epoxycarotenoid dioxygenases) (Figures S3 and S4; Table S5). These results suggest that the changes in flavonoid accumulation induced by melatonin might be related to crosstalk between melatonin and other plant hormones. Extensive research indicates that melatonin can regulate the synthesis and metabolism of many plant hormones and participate in hormone signaling transduction by recognizing hormone response elements [15,16]. Cis-element analysis showed that the 21 TFs contained five kinds of common hormone response elements in the promoter (Figure 5B), suggesting that these TFs might be recognized by melatonin signaling and thus underwent changes in transcript expression levels. However, the specific mechanisms need to be further explored. Three TFs also contain circadian control elements in their promoters, implying that circadian oscillators might also influence their expression.

Melatonin, first discovered in the bovine pineal gland, is an important hormone involved in the regulation of the circadian clock and sleep in animals [47]. The circadian oscillator in plants can similarly regulate the phases of various biological processes, including photosynthesis, flowering, yield, secondary metabolism, and biotic/abiotic stresses, by responding to light–dark cycles. Melatonin may act as a regulatory molecule in plant circadian rhythms and photoperiod-related changes [14]. Kolář et al. [48] observed fluctuations in melatonin levels during a 12/12 light–dark cycle in *Chenopodium rubrum*, but no correlation was found between different photoperiodic cycles and increased melatonin, implying that melatonin biosynthesis is not directly regulated by light, but more related to rhythms. Currently, the fluctuation in melatonin levels with circadian rhythms has been observed in many plants, including *Vitis vinifera* [49], *Prunus avium* L. [50], and *Malus zumi* [51]. Unlike in mammals, melatonin levels in plants do not always peak at night, and the fluctuations differ across plants [14]. However, the specific molecular mechanisms through which melatonin regulates plant circadian rhythm changes have not been revealed. The specific patterns of endogenous melatonin fluctuations with circadian rhythms in citrus fruits have yet to be reported. This study showed that melatonin can affect the core genes of the circadian oscillator in citrus, including *CCA1/LHY1*, and post-transcriptionally modify CCA1 and TOC1 proteins by up-regulating *CSNK2* and *ZTL*. This result provided some insights for understanding the specific molecular mechanisms underlying melatonin regulation of circadian rhythms.

Changes in the core oscillator would cause rhythm-related alterations in the photosynthesis system, flowering, and flavonoid biosynthesis pathway. Many studies have reported diurnal variations in flavonoid accumulation [52,53]. The flavonoid pathway is also regulated by the core circadian oscillator, with the transcription factor *PAP1* and a series of structural genes in the pathway, such as *CHS*, exhibiting circadian fluctuant changes [33]. Correspondingly, a deficiency in flavonoids led to altered expression of core oscillator genes in plants. The transcriptional activities of *CCA1* and *TOC1* were changed in *CHS*-deficient Arabidopsis, with an increase in *CCA1* expression levels but a decrease in *TOC1*. The same pattern existed in *F3’H*-deficient lines, suggesting that flavonoids with B-ring dihydroxylation may directly affect the transcription and expression of circadian rhythm genes [54]. Interestingly, since the hydroxyl group on the B-ring of citrus PMFs is almost *O*-methylated, further in-depth research is needed to determine whether PMFs affect circadian rhythm-related genes in citrus.

Although both *CHS* and *CAB* are regulated by the core oscillator, the rhythmic fluctuations in *CHS* and *CAB* in Arabidopsis show different phases and amplitudes, suggesting that they are subjected to distinct circadian clocks [34]. Here, *CHS* gene expression was significantly down-regulated, but *SIG5* and a range of *CAB* genes were up-regulated by melatonin treatment, consistently with other studies on melatonin regulation of the photosynthetic system. Extensive research has reported that melatonin improves photosynthesis in plants under stress, enhances chlorophyll content and photosynthetic efficiency, up-regulates genes related to photosynthesis and CO_2_ fixation, and protects chloroplasts from damage [55,56,57,58]. Melatonin can also increase the chlorophyll content in citrus under salt stress, protecting the citrus photosynthetic system [59]. Given that flavonoids are also important photo-protectants in plants, it can be speculated that exogenous melatonin could modulate the circadian rhythms to increase the protection of the photosynthesis system while affecting the flavonoid biosynthesis pathway through multilevel cascading regulation, resulting in differential changes in flavonoid contents.

In addition, transcriptomic and metabolomic data revealed that after melatonin treatment, a large number of DEGs related to terpenoid pathways and lipid-related processes were enriched, including “cutin, suberine, and wax biosynthesis” (Figure 3E). The abundance of some nonvolatile terpenoids was also changed (Figure S4, Table S1). A study on finger citron (*Citrus medica* L.) has found that melatonin treatment could increase bicyclogermacrene content and the expression level of *TPS1* [60]. Wang et al. [61] found that exogenous melatonin treatment in litchi (*Litchi chinensis* Sonn.) retarded fruit browning by reducing changes in cell membrane permeability during storage and inhibiting the increase in the activities of phospholipase D, lipase, and lipoxygenase. The findings in this study suggest that exogenous melatonin not only changed the accumulation of flavonoids but also affected other characteristic metabolites in citrus fruit peel. These results will provide a perspective for further studying the relationship between melatonin and citrus fruit quality formation.

## 4. Materials and Methods

### 4.1. Plant Materials and Melatonin Treatment

The citrus “Bingtangcheng” fruit (*Citrus sinensis* (L.) Osbeck) was cultivated in an orchard in Quzhou, Zhejiang province, China, and harvested 200 days after flower blooming. Fruits of uniform size, without disease or mechanical injuries, were selected for subsequent treatment.

Fruit samples were divided into four groups and treated with melatonin at the four different concentrations of 0.05 mmol L^−1^, 0.1 mmol L^−1^, 0.2 mmol L^−1^, and 0.5 mmol L^−1^, respectively. Each group contained six fruits, with each fruit as a biological replicate. The detailed treatment method was as follows: A volume of 5 mL melatonin solution (as treatment) or ddH_2_O (as control) was injected into the peel on opposite sides of the equatorial plane of each fruit. After injection, the fruits were wiped to dry them and then stored at 12 °C, RH 80%, in the dark to avoid the decomposition of melatonin. After seven days of storage, the treated part (a circle of area 2πr^2^ cm^2^) was separated from the fruit, then chopped, instantly frozen with liquid nitrogen, and stored at −80 °C until use.

### 4.2. Chemicals

Ten flavonoid standards were used for the qualitative and quantitative analyses of the total flavonoid extract. Narirutin, hesperidin, didymin, isosinensetin, sinensetin, nobiletin, tangeretin, and 5-demethylnobiletin were purchased from Yuanye Biotechnology Co., Ltd. (Shanghai, China). 8-Demethoxytangeretin and heptamethoxyflavone were purchased from SinoStandards Bio-Tech (Chengdu, China). Acetonitrile and methanol of chromatographic grade for HPLC were purchased from Sigma-Aldrich (St. Louis, MO, USA). Melatonin powder of BC grade for treatment was purchased from Sangon Biotech (Shanghai, China).

### 4.3. Flavonoid Extraction and HPLC Analysis

Total flavonoids were extracted and analyzed by HPLC according to the previous report [29]. The fruit samples were ground into a fine powder in liquid nitrogen, and 0.5 g of powder from each sample was ultrasonically extracted with 3 mL of 80% ethanol (*v/v*) for 30 min. Then, the supernatant was collected by centrifugation at 5000 rpm for 10 min. Extraction was repeated three times, and all the supernatants (9 mL) were combined and taken as the total flavonoid solution. A volume of 500 μL of total flavonoid solution was centrifuged at high speed (>12,000 rpm) for 20 min to remove impurities, and the supernatant was added to a sample vial to be injected into a Waters HPLC system (Waters Corp., Milford, MA, USA). 

Chromatographic separation was conducted by utilizing a Sunfire C18 ODS column (4.6 × 250 mm, 5 μm, Waters Corp.) at room temperature and a flow rate of 1 mL min^−1^. The mobile phases of 0.1% formic acid–water (*v/v*) (solvent A) and acetonitrile (solvent B) were utilized with a linear gradient program as follows: 0 min/20% B, 5 min/20% B, 10 min/27% B, 15 min/27% B, 25 min/40% B, 35 min/60% B, 40 min/80% B, 42 min/100% B, 45 min/20% B, and 50 min/20% B. Flavanone glycosides were monitored at 280 nm, while PMFs were monitored at 330 nm. The flavonoids were quantified by comparing the peak area to the standard curves.

### 4.4. Nontargeted Metabolome Detection

The metabolites of the samples were extracted, detected, and analyzed by BGI Genomics (Shenzhen, China) according to their standard procedures. A total of 50 μg of sample powder was soaked with 800 μL of pre-cooled 70% methanol (*v/v*) and 20 μL of internal standard (d_3_-Leucine, ^13^C_9_-Phenylalanine, d_5_-Tryptophan, and ^13^C_3_-Progesterone) in 1.5 mL EP tubes and mixed thoroughly. The mixture was sonicated at 4 °C in a water bath for 30 min and subsequently allowed to stand at −20 °C for 1 h. Then, the extracts were centrifuged at 14,000 rpm and 4 °C for 15 min, and the supernatants were collected and filtered through a 0.22 μm membrane prior to LC-MS/MS analysis. A volume of 20 μL of filtered supernatant from each sample was mixed, and the solution was taken as the QC (quality control) sample to assess the reproducibility and stability of the LC-MS/MS analysis (Figure S5).

The separation and detection of the metabolites were performed on a Waters 2777C UPLC system (Waters Corp., Milford, MA, USA) in tandem with a Q Exactive HF high-resolution mass spectrometer (Thermo Fisher Scientific Inc., Waltham, MA, USA), equipped with a Hypersil GOLD αQ Dim column (2.1 × 100 mm, 1.9 μm, Thermo Fisher Scientific). The mobile phases were 0.1% formic acid–water (*v/v*) (solvent A) and 0.1% formic acid–acetonitrile (solvent B) with a linear gradient program as follows: 0 min/5% B, 2 min/5% B, 22 min/95% B, 27 min/95% B, 27.1 min/5% B, and 30 min/5% B, at a flow rate of 0.3 mL min^−1^. Mass spectrum conditions were as follows: The scanning ranges (*m*/*z*) were 125–1500 Da for positive ion mode and 100–1500 Da for negative ion mode with a resolution of 120,000 in MS^1^, and the automatic gain control targets for MS acquisitions were set to 1 × 10^6^ (positive ion mode) and 3 × 10^6^ (negative ion mode) with a maximum injection time of 100 ms. The top 3 parent ions with the highest intensity were selected for subsequent MS^2^ fragmentation with a maximum injection time of 50 ms and a resolution of 30,000. The AGC was set to 2 × 10^5^ (positive ion mode) and 1 × 10^5^ (negative ion mode), and the stepped normalized collision energy was set to 20, 40, and 60 eV. ESI conditions were set as follows: sheath gas flow rate, 40; auxiliary gas flow rate, 12; spray voltage, 3.8 |KV| for positive ion mode and 3.2 |KV| for negative ion mode; capillary temperature, 320 °C; auxiliary gas heater temperature, 350 °C.

The raw data from LC-MS/MS were imported into Compound Discoverer 3.3 (Thermo Fisher Scientific) software and analyzed in conjunction with the BMDB (BGI metabolome database), mzCloud database, and ChemSpider online database according to the following parameters: mass deviation of parent ions < 5 ppm; mass deviation of fragment ions < 10 ppm; retention time deviation < 0.2 min. The identified metabolites were functionally annotated by using the KEGG and HMDB (Human Metabolome Database) databases. The data matrix containing the identified metabolite information is listed in Table S1.

### 4.5. Metabolomic Data Analysis and Plot

Twelve samples were subjected to principal component analysis based on metabolite abundance and plotted by using the R package “princomp” embedded in the online platform OmicStudio tool (https://www.omicstudio.cn/tool, accessed on 6 June 2024). The average abundance (mean) and the standard deviation (SD) of each metabolite in each group of samples were calculated; then, the fold change (FC) of each metabolite in the comparison group “melatonin treatment vs. control (MLT vs. CK)” was determined. Student’s *t*-test was used to test the significance of the variation in metabolite abundance in the comparison group “MLT vs. CK”, and the *p*-value was used to assess the significance level of the difference in metabolite abundance between the two groups of samples. Metabolites with abundance FC > 1.2 or < 0.83 and *p*-value < 0.05 were defined as DAMs. A volcano plot was drawn by using the R package in the online OmicStudio tool based on the above threshold values.

### 4.6. RNA Extraction and Reverse Transcription Quantitative PCR (RT-qPCR)

Total RNA of citrus fruit was extracted with a modified CTAB method described in a previous report [62]. A PrimeScript™ RT reagent Kit with gDNA Eraser (Takara, Dalian, China) was used to eliminate the residual genomic DNA from total RNA and synthesize the cDNA strands for RT-qPCR via reverse transcription. To validate the gene expression profiles, RT-qPCR was performed as described before [31] on a Bio-Rad CFX96 instrument (Bio-Rad, Hercules, CA, USA) by using a TB Green Premix Ex Taq (Tli RNaseH Plus) kit (Takara, Dalian, China). Gene-specific primers are listed in Table S6, and their specificity was confirmed by melting curves and PCR product re-sequencing. The relative abundance of gene transcripts was calculated by the 2^−ΔCT^ method, with citrus *β-Actin* serving as a housekeeping gene.

### 4.7. RNA Sequencing

The RNA sequencing (RNA-seq) of citrus fruit with 0.1 mmol L^−1^ melatonin treatment was conducted by BGI Genomics (Shenzhen, China) on the DNBSEQ platform following their standard procedures. A total of 12 samples were sequenced in this project, yielding an average of 6.41 Gb of data per sample. The average mapping rate of the samples to the reference genome was 94.12%. The raw data were filtered by removing the adapter contamination, reads containing an unknown base ratio >10%, and low-quality reads. Then, the clean data were mapped to the *Citrus clementina* reference genome (http://www.citrusgenomedb.org/species/clementina/genome1.0, accessed on 6 June 2024) by HISAT (v2.1.0) and mapped to the assembled unique gene by Bowtie2 (v2.2.5). The expression levels of all genes were calculated by RSEM (v1.2.8) and normalized to fragments per kilobase of transcript per million mapped reads (FPKM). The assembled unigenes were functionally annotated with KEGG, GO, and NR databases, and the TFs were predicted.

### 4.8. Transcriptomic Data Analysis and Plot

DEGs between melatonin treatment and control groups were obtained by using DESeq2 in TBtools-II (v2.069) [63] with the cutoff threshold of |log_2_(fold change)| ≥ 1 and FDR < 0.05. The PCA analysis and volcano plot of transcriptomic data of twelve samples were drawn as described above in Metabolomic Data Analysis. The Pearson correlation coefficients of transcriptomic data between different samples were calculated and plotted using the R package “corrplot” in the online OmicStudio tool. The samples were sorted by utilizing the AOE algorithm in the figure. DEGs were further functionally enriched according to the annotation and classification of KEGG pathways by using the online OmicStudio tools, and the significantly enriched pathways or entries (with *p*-value < 0.05) were displayed by R package ggplot2.

### 4.9. Integrated Analysis of Metabolomic and Transcriptomic Data

The DAFs were subjected to Pearson correlation analysis with the DEGs in the flavonoid biosynthesis pathway (including 11 differentially expressed *FOMTs* with a *p*-value < 0.5 < FDR) and all differentially expressed TFs. |Pearson correlation coefficients (PCC)| > 0.8 and *p* < 0.05 were set as threshold values of significant correlations. The top 5 positively correlated genes and the top 5 negatively correlated genes of each DAF with the highest |PCC| values were selected as co-expressed genes. If fewer than five genes met the threshold for significant correlations of a DAF, all correlated genes were retained. Four DAFs are not shown due to the absence of the genes that met the significant correlation threshold. The eight candidate genes of the flavonoid biosynthesis pathway were further subjected to Pearson correlation analysis with the 21 candidate TFs, and |PCC| > 0.8 and *p* < 0.05 were also set as the threshold of significant correlations. The co-expression network of DAFs and flavonoid pathway genes, as well as TFs, were visualized by Cytoscape (v3.10.1). The cis-element analysis and visualization of candidate TF promoters were performed by TBtools-II (v2.069).

### 4.10. Statistical Analyses and Plots

Statistical analysis was conducted by using paired Student’s *t*-test. IBM SPSS Statistical 19 (IBM Corporation, Armonk, NY, USA) was used for significance analysis with a confidence level of 95.0% (* *p* < 0.05), 99.0% (** *p* < 0.01), or 99.9% (*** *p* < 0.001). All experiments were performed with six biological replicates, and the data were presented as means ± standard error (SE). Graphpad prism 8 (GraphPad Software, San Diego, CA, USA) was used to plot figures other than omics data. ChemBioDraw Ultra version 20.0 (PerkinElmer Informatics, Waltham, MA, USA) was used to generate chemical structural formulas.

## 5. Conclusions

In summary, melatonin at a concentration of 0.1 mmol L^−1^ had a significant effect on citrus flavonoids, leading to an increase in PMF contents and a decrease in the content of other flavonoids, including flavone glycosides and flavonol glycosides. Numerous genes in the phenylpropanoid pathway were differentially expressed, with the transcript expression levels of *4CL*, *FNS*, and most *HCTs* being up-regulated and *CHS* being down-regulated, consistently with flavonoid abundance changes. After melatonin treatment, the expression of many AP2-EREBP TFs was altered, and eight of them were significantly co-expressed with DAFs. In addition, multiple genes related to circadian rhythm and photosynthesis were differentially expressed. Taken together, the effects of melatonin on citrus flavonoids might be mediated through multiple regulatory mechanisms, including direct regulation, hormone crosstalk, and influence that draw on the circadian rhythms. Moreover, melatonin also affected the abundance of other major metabolites in citrus fruit peel. The present study provides insights into the function of melatonin in citrus flavonoid metabolism and broadens the biological function of melatonin in citrus fruits.

## Figures and Tables

**Table 1:** Information of 21 TFs in the co-expression network.

Gene ID	TF Family	No. of Correlated DMAs	No. of Co-Expressed DEGs	No. of Co-Expressed Pathway Genes	No. of Hormone Response Elements in Promoters	Expression Level (FPKM)		
						CK	MLT	Fold Change (MLT vs. CK)
Ciclev10021265m.g	AP2-EREBP	5	11	3	6	18.58 ± 4.00	115.87 ± 28.62	4.96
Ciclev10005570m.g	AP2-EREBP	1	9	3	6	47.84 ± 7.35	185.84 ± 41.79	3.02
Ciclev10026052m.g	AP2-EREBP	4	6	0	1	15.90 ± 2.04	6.40 ± 1.38	0.42
Ciclev10026032m.g	AP2-EREBP	3	15	4	25	9.77 ± 1.51	3.65 ± 1.13	0.44
Ciclev10026602m.g	AP2-EREBP	9	11	5	13	31.70 ± 7.52	14.31 ± 1.63	0.44
Ciclev10025985m.g	AP2-EREBP	1	9	3	4	2.23 ± 0.54	9.76 ± 1.95	3.43
Ciclev10023546m.g	AP2-EREBP	1	12	5	11	94.18 ± 12.84	20.62 ± 11.16	0.28
Ciclev10010237m.g	AP2-EREBP	1	7	5	3	0.84 ± 0.13	0.35 ± 0.06	0.47
Ciclev10016289m.g	bHLH	2	11	3	9	1.66 ± 0.29	7.63 ± 1.87	3.53
Ciclev10030052m.g	bHLH	1	12	3	12	1.10 ± 0.20	3.64 ± 0.70	2.46
Ciclev10003771m.g	C2C2-GATA	11	11	5	4	3.23 ± 0.94	0.94 ± 0.26	0.34
Ciclev10032922m.g	C2H2	6	9	4	12	20.20 ± 4.26	8.08 ± 2.65	0.49
Ciclev10032889m.g	C2H2	3	12	4	10	366.58 ± 67.42	126.06 ± 49.14	0.47
Ciclev10018041m.g	E2F-DP	1	15	5	1	2.13 ± 0.19	0.88 ± 0.23	0.45
Ciclev10013705m.g	LOB	8	11	4	13	169.44 ± 37.39	67.18 ± 9.61	0.39
Ciclev10009286m.g	MYB	3	8	4	10	1.67 ± 0.58	0.63 ± 0.19	0.47
Ciclev10015976m.g	NAC	5	11	4	11	11.68 ± 1.08	5.17 ± 0.86	0.43
Ciclev10013812m.g	OFP	1	17	5	8	2.77 ± 0.20	1.17 ± 0.33	0.50
Ciclev10031289m.g	Sigma^70^-like	4	11	3	20	0.86 ± 0.12	2.35 ± 0.41	2.11
Ciclev10013486m.g	Trihelix	3	3	0	3	16.81 ± 3.87	6.36 ± 0.89	0.39
Ciclev10019820m.g	WRKY	3	10	3	18	3.50 ± 0.54	11.58 ± 2.20	2.67

## Data Availability

The original contributions presented in the study are included in the article/supplementary material, and further inquiries can be directed to the corresponding author.

## Supplementary Materials

The following supporting information can be downloaded at: https://www.mdpi.com/article/10.3390/ijms25126632/s1.
