# Supplementary material for: Integrated Metabolomic–Transcriptomic Analyses of Flavonoid Accumulation in Citrus Fruit under Exogenous Melatonin Treatment
Source: Int J Mol Sci. 2024 Jun 16;25(12):6632. doi: 10.3390/ijms25126632 (PMC11204001; doi:10.3390/ijms25126632)
Supplement: Supplementary file 1 [file ijms-25-06632-s001.zip › Supplementary Figures-revision.pdf]

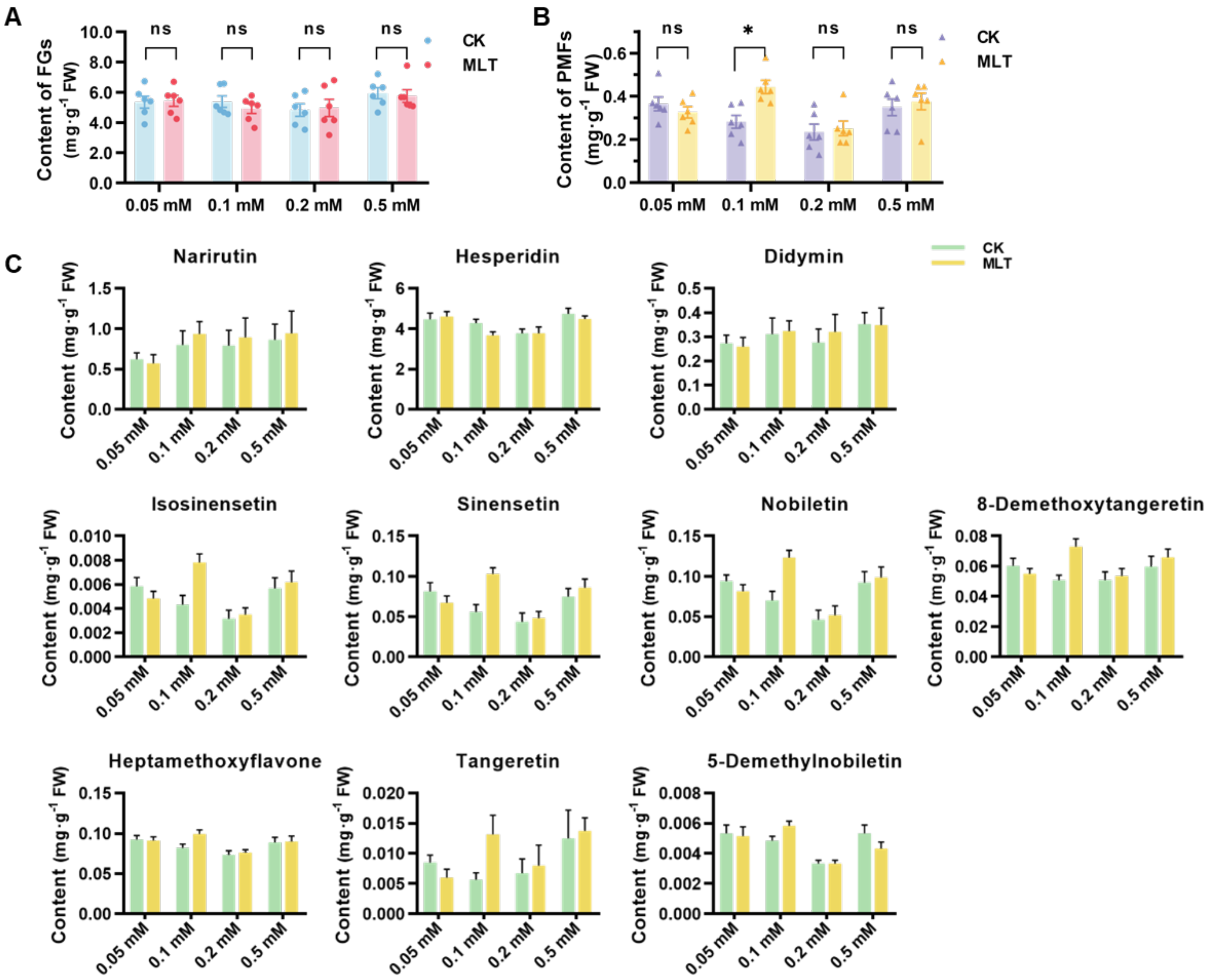

Figure S1 Effect of different concentrations of melatonin on citrus flavonoid contnts.

(A-B) Changes of total FG (flavanone glycoside) contents (A) and total PMF contents (B) in fruit peel after different concentrations of melatonin treatment. (C) Changes of individual flavonoid content. CK, control group; MLT, melatonin treatment group; FW, fresh weight. Error bars represent SE (n=6).

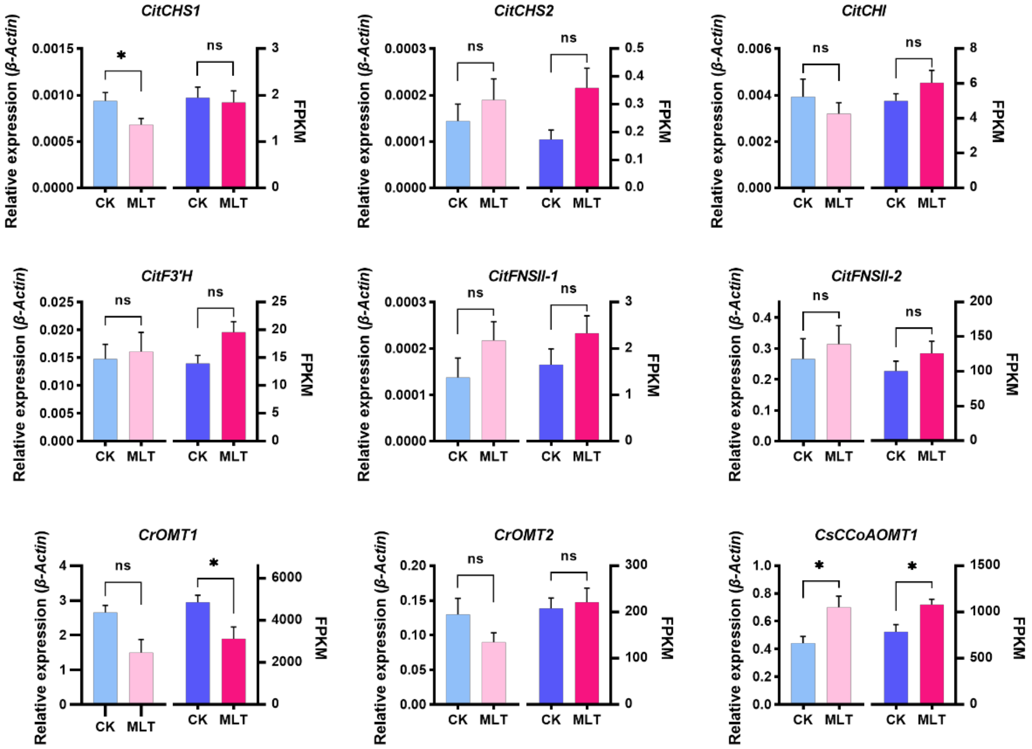

Figure S2 Expression profiles of the flavonoid biosynthesis pathway genes verified by RT-qPCR.
Error bars represent SE (n=6). Statistics analysis: Paired student's *t*-test (* $P < 0.05$). ns, not significant.

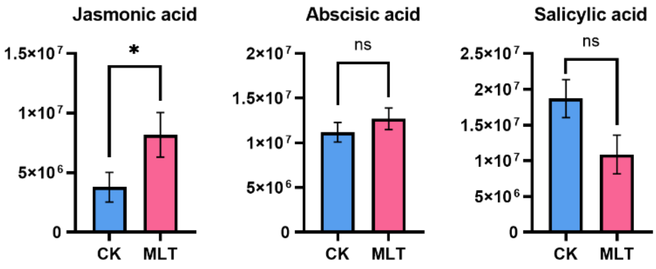

Figure S3 Abundance changes of three plant hormones detected in metabolomics.
Error bars represent SE (n=6). Statistics analysis: Paired student's *t*-test (* $P < 0.05$).

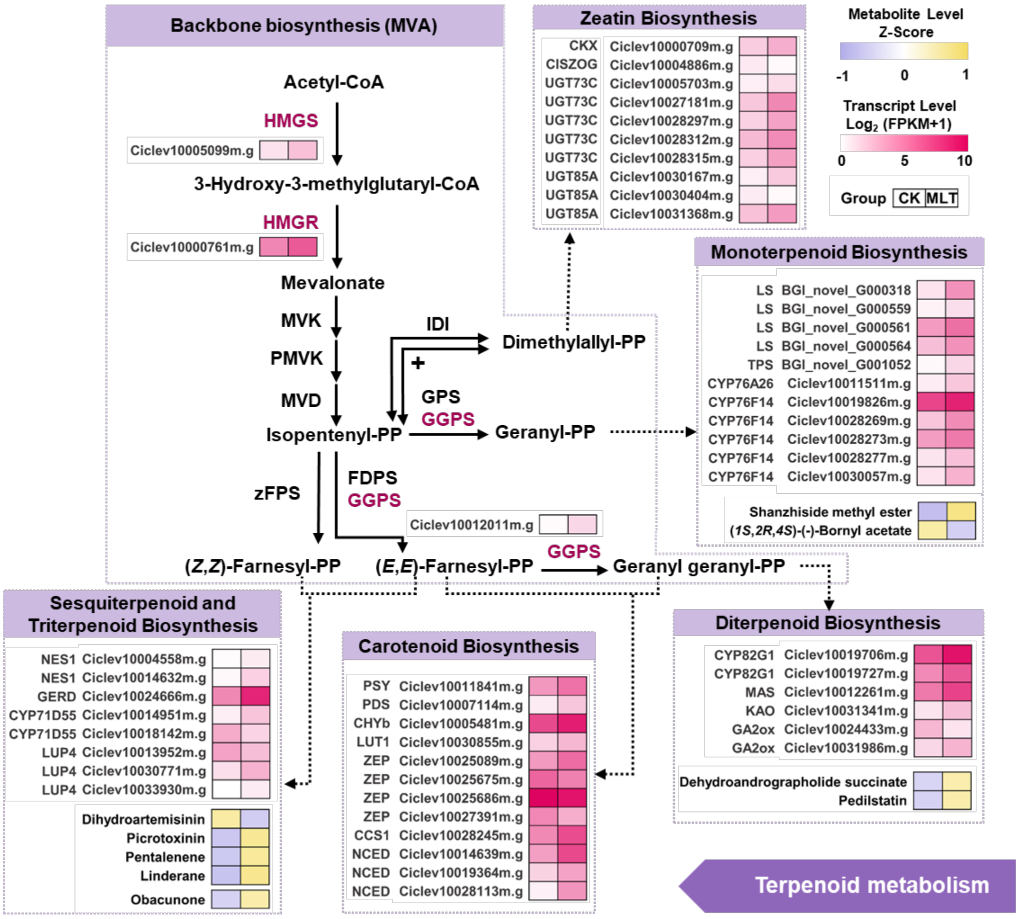

Figure S4 Heatmap of DEGs and DAMs in terpenoid metabolism pathway.

A

B

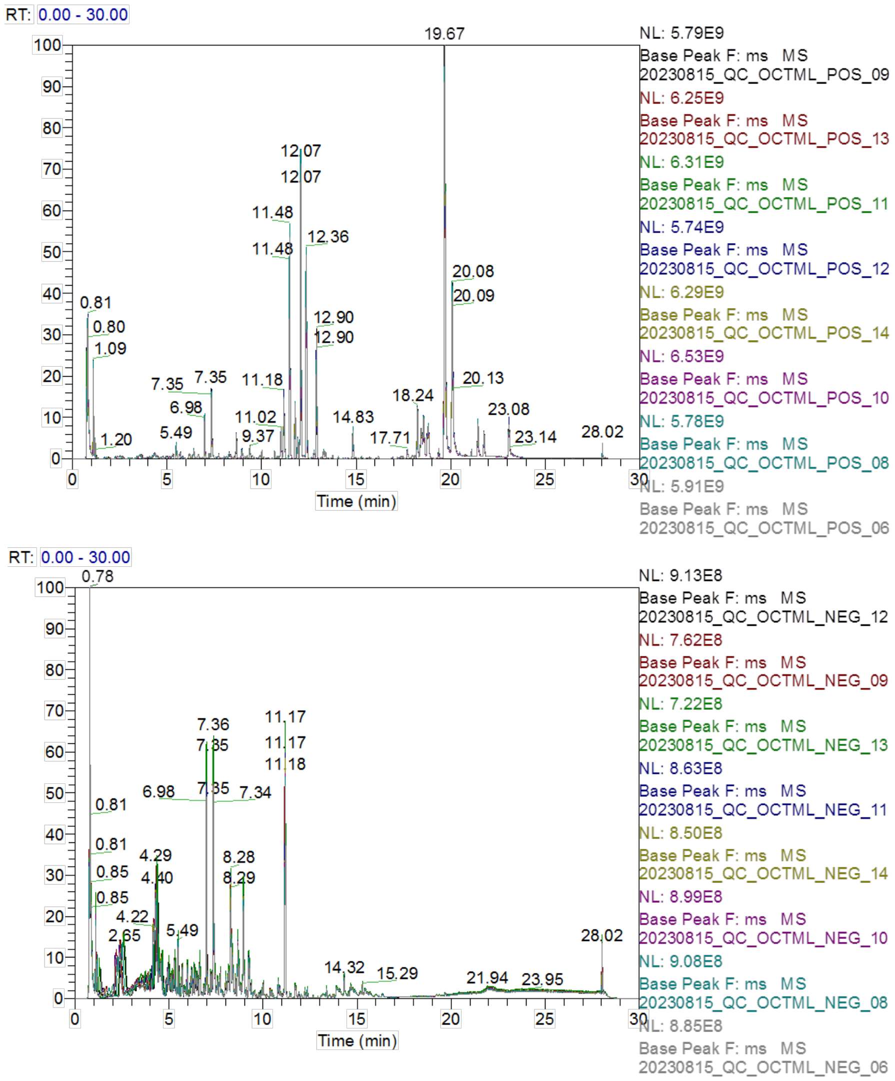

Figure S5 Base peak chromatograms of QC samples detected by UPLC-MS/MS.
(A) Positive ion mode. (B) Negative ion mode.
